# Trained immunity as a possible newcomer in autoinflammatory and autoimmune diseases pathophysiology

**DOI:** 10.3389/fmed.2022.1085339

**Published:** 2023-01-10

**Authors:** Anne-Sophie Beignon, Caroline Galeotti, Mickael M. Menager, Adrien Schvartz

**Affiliations:** ^1^Center for Immunology of Viral, Auto-immune, Hematological and Bacterial Diseases/Infectious Diseases Models and Innovative Technologies (IMVA-HB/IDMIT), U1184, Université Paris-Saclay, INSERM, CEA, Fontenay-aux-Roses, France; ^2^Department of Pediatric Rheumatology, Reference Center for AutoInflammatory Diseases and Amyloidosis (CEREMAIA), Hôpital Bicêtre, AP-HP, Le Kremlin-Bicêtre, France

**Keywords:** innate immunity, trained immunity, autoinflammatory diseases, autoimmune disease, pathophysiology

## Abstract

Autoimmune disorders have been well characterized over the years and many pathways—but not all of them–have been found to explain their pathophysiology. Autoinflammatory disorders, on the other hand, are still hiding most of their molecular and cellular mechanisms. During the past few years, a newcomer has challenged the idea that only adaptive immunity could display memory response. Trained immunity is defined by innate immune responses that are faster and stronger to a second stimulus than to the first one, being the same or not. In response to the trained immunity inducer, and through metabolic and epigenetic changes of hematopoietic stem and progenitor cells in the bone marrow that are transmitted to their cellular progeny (peripheral trained immunity), or directly of tissue-resident cells (local innate immunity), innate cells responsiveness and functions upon stimulation are improved in the long-term. Innate immunity can be beneficial, but it could also be detrimental when maladaptive. Here, we discuss how trained immunity could contribute to the physiopathology of autoimmune and autoinflammatory diseases.

## Introduction

For years, it has been believed that only adaptive immunity can develop faster and stronger responses to a second encounter with the same antigen by acquiring immune memory (1). Immune memory involves specific B and T lymphocytes and takes days to develop. It is the basis of vaccines. However, immune memory may not be so exclusive to the adaptive system. First introduced in 2011, the term “trained immunity” encompasses the innate immune system capacity to mount a more robust and faster response to a later stimulus after an effector response to an initial stimulus followed by a return to a non-activated resting state (2). In contrast to B and T cell immune memory based on clonal expansion, contraction, and differentiation, as well as BCR and TCR gene recombination, trained immunity relies on metabolic and epigenetics modifications to ensure improved and enhanced, but non-specific, innate responses. This innate memory has helped plants and invertebrates, which lack an adaptive immune system, survive infections throughout evolution (3–5). As a relatively new field of research, trained immunity still holds many secrets. But as in every system, balance is key. And an imbalance in what has helped life prosper might also be responsible for diseases mediated by the immune system, namely, autoinflammatory and autoimmune diseases. Autoimmune diseases are defined by auto-reactive T and B cells responsible for attacking auto-antigens and generating autoantibodies (6). Autoimmune diseases can be organ-specific such as type 1 diabetes or autoimmune thyroiditis, but they can also be more systemic such as systemic lupus erythematosus (SLE). On the other hand, autoinflammatory diseases are defined by flares of systemic inflammation without apparent involvement of antigen-specific T cells or significant production of autoantibodies (7). Prototypic autoinflammatory diseases are hereditary recurrent fevers like familial Mediterranean fever (FMF) and mevalonate kinase deficiency (MKD) (8). However, the limit between autoimmune and autoinflammatory diseases is not so clear and the frontier between the two definitions is blurrier than ever (9). Adaptive immunity has been studied for a long time. In comparison, fewer studies have been solely focused on innate immunity and innate cells such as monocytes, neutrophils, or natural killer (NK) cells, and even less on innate immune memory (10, 11). Hence, our knowledge of the two immune systems and their interactions is fragmented. New technologies such as single-cell gene expression analyses (12, 13) and high dimensional cytometry, being spectral or mass cytometry (14, 15), have allowed the exploration of new pathways and deeper phenotyping of cells and characterization of cell subsets and state of maturation/activation. An even wider approach consists of systems immunology with the integration of omics technologies data and gives an even more precise data-driven idea of what is really going on *in vivo* (16).

In this review, we will discuss whether trained immunity could play a role in the pathophysiology of immune-related diseases.

## Physiological basis of trained immunity

**FIGURE 1:**
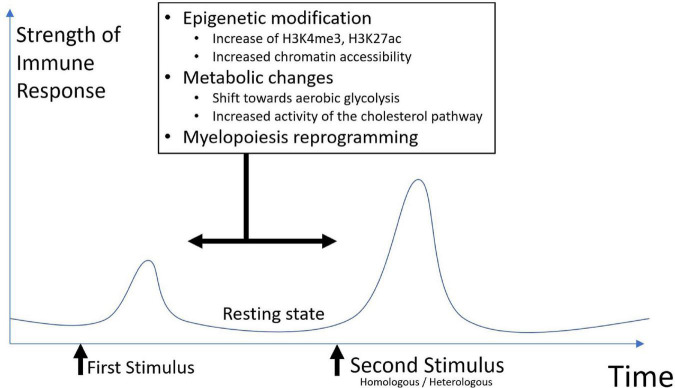
Basic representation of trained immunity.

Trained immunity is characterized by metabolic and epigenetic changes. These changes will generate an altered innate response to a second stimulus. The response will be more robust, and faster but non-specific (Figure 1).

### Innate system components involved in trained immunity

The innate immune system ensures a first immune response against microorganism exposure and infection or tissue injury. Its principal components are circulating molecules like cytokines and complement, and cells of hematopoietic origin like neutrophils, macrophages, dendritic cells, NK cells as well as tissue-resident cells of embryonic origin such as alveolar macrophages in the lungs or Kupffer cells in the liver (17–19).

Innate cells express pattern recognition receptors (PRR) that recognize pathogen-associated molecular patterns (PAMPs) or damage-associated molecular patterns (DAMPs). Those receptors include Toll-like receptors (TLRs) located on cell surfaces or in the membrane of intracellular vesicles. Other PRRs include–but not only–the nucleotide-binding oligomerization domain (NOD) like receptors (NLRs), RIG-like receptors, and C-type lectin receptors. Once activated, PRRs trigger a cascade of activation that leads to a secretion of a wide range of inflammatory and antiviral cytokines such as interleukin (IL) 1β, IL6, C-X-C Motif Chemokine Ligand 8 (CXCL8), IL12, Tumor Necrosis Factor (TNF)α and interferon (IFN) type I. IL1β, IL6, and TNFα have been shown to be involved in trained cells induction (20–22). PRR triggering also results in various innate effector functions other than cytokine production, such as phagocytosis, neutrophil extracellular traps (NET), reactive oxygen species (ROS) production, or pathogen killing among others (23–26). This innate/inflammatory response is strictly regulated and leads normally to potent tissue repair, dead cells and pathogen elimination, and a rapid return to a steady and resting state once inflammation is resolved. Depending on the nature of the initial stimulus, trained cells can be induced, and more or less time after the first stimulus, they can respond differently to a second homologous or heterologous stimulus than naïve innate cells.

Monocytes and neutrophils can be trained, however, they have a short lifespan. Patel et al. showed that classical monocytes CD14++ CD16- have approximately a 1 day life span, intermediate monocytes CD14++ CD16+ have a longer lifespan of ∼4 days, and non-classical monocytes (CD14+ CD16+) have a ∼7 days lifespan after activation (27). Neutrophils are even more short-lived (28). This conundrum was solved by the demonstration that trained immunity generates a long-lasting memory through metabolic changes—glycolysis, cholesterol pathways modifications—in hematopoietic stem and progenitor cells (HSPCs) in the bone marrow (BM) *via* a type II interferon (IFN-g) or IL1β response mediated mechanism after Bacillus Calmette-Guerin (BCG) or b-glucan (which represent two canonical trained immunity inducer) systemic administration (29). Hence, in mice injected intravenously with BCG, the BM-derived macrophages were trained and showed better protection against tuberculosis, even if they had not encountered BCG themselves (30).

### Metabolic changes

Metabolic rewiring is a key feature of trained immunity. Different pro-inflammatory cytokines like IL1β, IL6, and TNFα as well as PRRs ligands can trigger different metabolic pathways in monocytes, which will, in turn, interact with the mechanistic target of rapamycin (mTOR), a master regulator of metabolism and immunity (31). The mTOR/Hypoxia-inducible factors (HIF)-1α/AKT pathway induces a shift toward aerobic glycolysis from oxidative phosphorylation when stimulated by β-glucan, a cell wall component of fungi and classic inductor of trained immunity (32). β-glucan-trained monocytes show elevated activity of the cholesterol synthesis pathway and increased level of mevalonate, both of which enhance the inflammatory response through an mTOR-mediated pathway (33). When monocytes are exposed to fumarate, a Krebs cycle metabolite, a decrease in the activity of Lysine-specific demethylase 5A (KDM5) and an increase in the methylation of histone lysine 4 residues (H3K4me3) at the promoters of the proinflammatory genes coding for IL6 and TNFα are induced, linking metabolism to epigenetic. Once restimulated with lipopolysaccharide (LPS) after a resting step, these *in vitro*-trained monocytes showed increased production of TNFα (34) as well as structural changes in mitochondria (35). On the other hand, itaconate, a metabolite that interacts with the Krebs cycle, has been shown to participate in immune tolerance and reduce inflammation by targeting succinate dehydrogenase (36).

### Epigenetic changes

The other key feature of trained immunity is epigenetic reprogramming. In unstimulated myeloid cells, chromatin configuration is compact, thus making access to proinflammatory genes by the transcriptional machinery difficult (37, 38). Two major actors of epigenetic change in trained immunity are the enrichment of histone 3 lysine 4 trimethylation (H3K4me3) mark and the K27 acetylation on histone 3 (H3K27ac) mark in the promoters of pro-inflammatory genes (39). These changes will modify chromatin accessibility. After the initial stimulation, the resting state is characterized by a partial return of chromatin to its original condensed state. Chromatin remains actually mildly condensed, thus allowing an enhanced and faster transcriptional response after a new stimulus (21). Other mechanisms than histone modifications have been reported to be involved in trained immunity including DNA methylation and long non-coding RNA (lncRNA) (40).

The coordinated and interconnected metabolic and epigenetic modifications induced by a first encounter allow cells to shift metabolism toward glycolysis and to have better access to “trainable” genes thanks to chromatin modifications. A second encounter with a non-specific stimulus will trigger a faster and stronger response. Although it might be beneficial in several contexts, such as vaccines providing non-specific protection or increasing the immunogenicity of subsequent unrelated vaccines, such enhanced responsiveness and response might be damaging, causing flares or prolonged activity for immune-related diseases.

## Monogenic autoinflammatory diseases and trained immunity

Autoinflammatory diseases are characterized by flares of inflammation without any autoantibodies detected or concomitant infection. They associate fever with a vast array of symptoms. Major symptoms include, but are not limited to, skin involvement, serositis, joint pain, and abdominal pain. Inflammation can be normal between flares but can also be reduced without going back to normal, with a persisting low-grade chronic inflammation. Prolonged inflammation in untreated or uncontrolled diseases can lead to amyloidosis (8). Autoinflammatory diseases are mostly driven by innate immune components, hence trained immunity could explain part of their pathophysiology. Monogenic autoinflammatory diseases are better characterized on the genetic level than polygenic autoinflammatory diseases, with known mutations in single genes. We will discuss first the possible implication of trained immunity in monogenic and then in polygenic autoinflammatory diseases. Trained immunity phenotypes are summarized in Table 1.

**TABLE 1:** Trained immunity phenotype associated with autoinflammatory and autoimmune diseases.

Disease	Metabolic change	Epigenetic change	Cells involved	Cytokines	Other
Cryopyrin-associated periodic syndrome (CAPS)	–	DNA demethylation is decreased in untreated patients (45)	Monocytes from patients exhibit higher levels of ROS and mitochondria alteration (42, 43)	Increase in IL1β and IL18 (42, 43) a decrease of IL6 and IL1RA (42, 43)	Itaconate blocks IL1β secretion from PBMCs of CAPS patients (48) β glucan-induced macrophages inhibit NLRP3 inflammasome activation (46)
Familial Mediterranean fever (FMF)	–	–	PBMCs, Monocytes	Increased secretion of IL6 and TNFα between flares Increased secretion of IL1α, IL1β, IL6, IL8, IL12, IL18, and TNFα than cells from healthy donors (50–54)	–
Mevalonate kinase deficiency (MKD)	Higher-expressed glycolysis-related genes, returned to normal after anti-IL1β (canakinumab) treatment (58)	Modifications in the H3K27ac marker (33)	PBMCs	Increased proinflammatory cytokines (IL1, IL6, TNFα) in an unstimulated state, and show an increase of release after stimulation by LPS than healthy donors (55)	–
Tumor necrosis factor receptor-associated periodic syndrome (TRAPS)	–	–	Monocytes	Overexpression of IL1β and IL1R1r compared to healthy controls (60) Downregulation of TGFβ and IFN type I and type II gene expression (60)	IL1-blockade treatment improves patients (61) Treatment with an IL1β inhibitor reduces overexpressed genes to levels comparable to healthy donors (62)
Chronic non-bacterial osteomyelitis	–	Diminished phosphorylation of histone H3 serine 10 (H3S10) (65)	Monocytes	Increased amounts of pro-inflammatory cytokines (IL1ß, IL6, TNFα) without stimulation and after stimulation (64, 65)	–
Behcet’s disease (BD)	–	Reduced methylation rate of TLR4 in BD patients (72)	Monocytes	Increased amounts of pro-inflammatory cytokines (TNFα, IL6, IL8) without stimulation and after stimulation by LPS (69, 70)	–
Sarcoidosis	Higher gene expression of genes involved in the cholesterol pathway (83)	–	Monocytes, PBMCs	Higher secretion of TNFα, IL1ß, IL6, IL10, IL12. (77–79)	Increased phagocytic activity (81)
Systemic lupus erythematosus (SLE)	mTOR modifications (94, 95)	DNA methylation modifications. (93) H3K4me3 modifications (90, 91)	Monocytes (85–88)	Higher secretion of TNFα (89)	Stronger myeloid signature from HSPCs of a mouse model of SLE (92) mTOR inhibitors have proven effective (96, 97)
Sjögren’s Syndrome	–	Reduced histone acetylation (104) DNA methylation modifications (105)	Monocytes Monocytes-derived dendritic cells	Higher secretion of IL6, IL8 (100–102)	Higher differentially expressed genes of inflammatory pathways (106)
Rheumatoid arthritis	Glycolysis shift (110–112).	DNA methylation after treatment (113)	Monocytes, macrophages	Higher secretion of IL1ß and IL6 (108, 109)	–

### Cryopyrin-associated periodic syndrome (CAPS)

Cryopyrin-associated periodic syndrome or NLRP3-associated autoinflammatory disease is a group of diseases defined by missense mutations of NLRP3 (41). The diseases spectrum varies from Familial Cold Autoinflammatory Syndrome (FCAS) to Muckle-Wells syndrome to Neonatal Onset Inflammatory Multisystem Disease (NOMID) also known as Chronic Infantile Neurological Cutaneous and Articular (CINCA) syndrome. The symptoms include neutrophilic-like skin rash, fever, conjunctivitis, and, in the more severe forms, central nervous system involvement with ear loss, joint involvement, and secondary amyloidosis.

Biologically, the consequences of the mutation are an increase in IL1β and IL18, and a decrease in IL6 and IL1RA (42, 43). Interestingly, the study by Carta et al. shows that unstimulated monocytes from CAPS patients have a rather mild elevation of ROS and mitochondria alteration, but, after stimulation by LPS, it increases more than monocytes from healthy controls. Cytokine production of IL1β is higher after stimulation, but other cytokine production like IL1RA and IL6 decreases faster than the control. As already discussed, IL1β secretion can trigger a trained immunity phenotype, with ROS and mitochondrial modification (44). More importantly, after a stimulated state, monocytes do not return to the initial steady state but instead to a resting state where they are more responsive to a second unrelated trigger. This could explain part of the differences between the patients and control in an unstimulated state.

Another interesting study shows that DNA demethylation is decreased in monocytes of untreated CAPS patients, enhancing proinflammatory genes, compared to healthy controls. But this methylation profile can be reversed with anti-IL1 treatment. Treated patients with anti-IL1 therapy show a similar profile to healthy controls. An explanation could be that IL1 blockade prevents the generation of a trained immunity phenotype in the monocytes of CAPS patients, thus reversing toward healthy controls (45). Another study made on monocytes of CAPS patients and macrophages generated from monocytes of the same patients showed that β-glucan, a known inducer of trained immunity, could have the ability to block NLRP3 inflammasome activation. Monocytes from patients were differentiated with either granulocyte-macrophage colony-stimulating factor (GM-CSF) or macrophage colony-stimulating factor (M-CSF) after a 24-h preincubation period with or without β-glucan. Monocytes derived with GM-CSF showed higher secretion of IL1β compared to healthy donors. IL1β secretion was significantly inhibited by the addition of β-glucan in either condition and comparable to the level of healthy donors (46).

As we already discussed, itaconate is an unsaturated dicarboxylic acid. It is synthesized from the decarboxylation of *cis*-aconitate, a Krebs cycle intermediate (47). Itaconate has been shown to produce an anti-inflammatory action by interacting with the Krebs cycle (36). In the study by Hooftmann et al. they showed that itaconate exerts its anti-inflammatory action through the blockade of the interaction between NLRP3 and the mitotic kinase NIMA-related kinase 7 (NEK7) thus preventing the activation of NLRP3 (48). This could be one more piece of evidence linking trained immunity to CAPS syndromes.

### Familial Mediterranean fever

Familial Mediterranean Fever is the most common hereditary recurrent fever. It manifests by flares of 12 h up to 3 days. It associates fever, acute serositis, joint pain (knees, hips, and ankles), abdominal pain, and skin rash (49).

In FMF patients, peripheral blood mononuclear cells (PBMCs) show more elevated secretion of IL6 and TNFα at baseline state (between flares). PBMCs and monocytes stimulated by LPS exhibit more secretion of IL1α, IL1β, IL6, IL8, IL12, IL18, and TNFα than cells from healthy donors (50–54). An increase in the secretion of proinflammatory cytokines is one of the hallmarks of trained immunity.

### Mevalonate kinase deficiency

Mevalonate Kinase Deficiency, is an autoinflammatory disease caused by a mutation in mevalonate kinase (55). Patients have recurrent fever flares with gastrointestinal involvement (abdominal pain, diarrhea, vomiting), arthralgia or even arthritis, lymphadenopathy, and skin lesions (56). Patients with mevalonic aciduria may have psychomotor retardation with a cerebellar syndrome related to cerebellar atrophy (57).

It has been shown that PBMCs of MKD patients release more proinflammatory cytokines (IL1, IL6, TNFα) in an unstimulated state, and show an increase in the release after stimulation by LPS (55). Bekkering et al., showed that monocytes from MKD patients display a trained immunity phenotype and that this mechanism is associated with modifications in the H3K27ac marker (33). In a transcriptome study, MKD patients were shown to have higher-expressed glycolysis-related genes, which returned to normal after anti-IL1β canakinumab treatment (58). The increase of pro-inflammatory cytokines, epigenetic modification, and glycolysis-shift point toward a trained immunity phenotype.

### Tumor necrosis factor receptor-associated periodic syndrome

Tumor Necrosis Factor Receptor-Associated Periodic Syndrome (TRAPS) is caused by a dominant mutation in TNF Receptor Super Family 1A (TNFRSF1A). Symptoms are fever flares from 5 days up to 3 weeks, it involves abdominal manifestations (peritonitis-like abdomen), skin manifestations, and oedematous, of various sizes with hazy edges. Its main complication is secondary amyloidosis (59).

Monocytes of TRAPS patients present an overexpression of IL1β and IL1R1 receptor compared to healthy controls, at baseline, without stimulation. On the other hand, TGFβ and IFN type I and type II gene expression is downregulated (60). Interestingly, TRAPS patients are responsive to IL1-blockade treatment, as shown by clinical studies and real-life experience (61). Torene et al. showed that treatment with canakinumab, an IL1β inhibitor, reduces overexpressed genes, including TNFRSF1A gene expression, and put them to levels comparable to healthy donors (62). These modifications underline a possible trained immunity implication.

## Polygenic autoinflammatory diseases and trained immunity

Polygenic autoinflammatory diseases do not have a clear genetic signature. However, there are hints of trained immunity phenotype that we will discuss here.

### Chronic non-bacterial osteomyelitis

Chronic Non-bacterial Osteomyelitis (CNO) or Chronic Recurrent Multifocal Osteomyelitis (CRMO) is a bone disorder caused by bone inflammation. Symptoms include bone pain, swelling, and sometimes redness. Other symptoms may include psoriasis and palmoplantar pustulosis, inflammatory bowel disease, and severe acne. Some patients also display symptoms compatible with spondylarthritis (63).

Monocytes from CRMO patients show increased amounts of pro-inflammatory cytokines (IL1ß, IL6, TNFα) at baseline without stimulation (64, 65) and after stimulation (65). It was also found that this overexpression was associated with diminished phosphorylation of histone H3 serine 10 (H3S10), an activating epigenetic mark (65). Both of which could be linked to trained immunity phenotype.

### Behcet’s disease

Behcet’s disease (BD) is a vasculitis that can affect both veinous and arterial vessels. It can manifest with eye involvement, genital and oral aphthous, thrombosis, joint involvement, skin manifestations, and neurological manifestations (66). Its exact physiopathology is unknown but monocytes are part of the explanation (67, 68). Monocytes of BD patients produce more proinflammatory cytokines like TNFα, IL6, and IL8, without stimulation and after stimulation by LPS (69, 70). Colchicine, also used in FMF, has been an efficient treatment for a long time (71).

A study looking at TLR4 and TLR2 gene promoters was conducted in Iranian patients with BD. They showed that mRNA of TLR4 was increased in BD-active patients compared to inactive and healthy controls. The methylation rate of the TLR4 gene was reduced in the active and inactive patients compared to healthy controls. The authors suggested that the methylation profile of TLR4 might be involved in the BD pathophysiology (72). Another study involving TLR4 in Duchenne Muscular Dystrophy in a mouse model showed that TLR4 is a regulator of trained immunity. They found that the modified phenotype of monocytes/macrophages is regulated by TLR4. They also showed metabolism changes, increased baseline production in proinflammatory cytokines, and epigenetic modifications (73). All these elements point toward a possible implication of trained immunity.

### Sarcoidosis

Sarcoidosis is a systemic disease characterized by non-caseating granulomas. It affects preferentially the lungs and lymph nodes but other organs can be involved such as the heart, the eyes, the skin, the joints, or the central nervous system (74). A juvenile form of sarcoidosis, Blau syndrome, is caused by NOD2/CARD15 mutations (75).

Monocytes of patients with sarcoidosis express more TLR2 and TLR4 (76). Monocytes of sarcoidosis patients show more production of IL6 compared to healthy controls (77). PBMCs of sarcoidosis patients have spontaneous secretion of proinflammatory cytokines (TNFα, IL12) compared to healthy controls. They display higher secretion of TNFα, IL6, IL10, and IL12 after stimulation by LPS or β-glucan (78), as well as IL1β (79). Monocytes of sarcoidosis patients show more oxygen radicals (80) and increased phagocytic activity (81) compared to healthy controls. A study using RNA-sequencing analysis of monocytes from sarcoidosis patients found that the expression of several genes involved in monocyte activation, inflammation, metabolic pathways (interestingly the cholesterol pathway), and oxidative phosphorylation were highly enhanced (82). The cholesterol pathway involvement is of interest as it is a key feature of mevalonate kinase deficiency physiopathology (83). The genetic background, associated with the increase in proinflammatory cytokines secretion, as well as metabolic involvement could suggest trained immunity.

## Autoimmune diseases and trained immunity

Autoimmune diseases are characterized by the presence of autoantibodies. They can be organ-specific or more systemic. Classically defined by adaptive immunity and T and B cells aberrations, some evidence also points to an implication of innate cells, to various degrees. We will discuss here how trained immunity traits are described in autoimmune diseases. Trained immunity phenotypes are shown in Table 1.

### Systemic lupus erythematosus

Systemic lupus erythematosus is the prototypic systemic autoimmune disease. It is defined by multiple autoantibodies associated with a systemic illness where any organ can be targeted. Its pathophysiology is complex and not fully understood (84). Despite being characterized by autoantibodies, there is growing evidence that monocytes/macrophages can be part of the SLE pathophysiology, as well as other innate immune cells like dendritic cells (85–88). Monocytes from SLE patients have a significantly more important TNFα secretion than monocytes from healthy donors without stimulation (89). PBMCs and monocytes of SLE patients have been shown to have H3K4me3 alterations, one of the hallmarks of trained immunity (90, 91). Epigenetic reprogramming was also found at the hematopoietic level in BM-derived HSPCs from diseased lupus mice with a strong myeloid signature. HSPCs of SLE patients with severe diseases also had changes toward myelopoiesis, confirming the data from the mouse model (92). Metabolic changes impacting DNA methylation/demethylation have been reported in lupus patients (93) and mTOR implications are also reported to be involved in SLE patients (94, 95). Inhibitors of mTOR like sirolimus and everolimus have proven effective in the treatment of lupus (96, 97). Metabolic regulation has been shown to be effective in a lupus mouse model and PBMCs from lupus patients to alleviate symptoms through an mTOR and NLRP3 regulation effect (98). All these metabolic and epigenetic reprogramming, as well as the cells involved, could be explained by trained immunity.

### Sjögren’s syndrome

Sjögren’s syndrome is a systemic autoimmune disease causing eye and mouth dryness, fatigue, and joint pain. It can be primary or associated with other autoimmune diseases (99). Monocytes of Sjögren’s syndrome patients showed increased proinflammatory cytokines production (TNFα, IL1, IL6) after stimulation (100–102). Increased proinflammatory cytokines (IL6, IL8) production was also found in monocyte-derived dendritic cells of primary Sjogren patients (103). Histone acetylation was reduced in PBMCs from Sjögren’s patients compared to healthy controls (104). Epigenetic modifications such as DNA methylation is also involved in the pathophysiology (105). Differentially expressed genes were enriched in the cellular and inflammatory response to cytokine in monocytes of Sjögren’s Syndrome patients and SLE patients compared to healthy controls (106). These different elements put together such as cell type, and proinflammatory production, associated with epigenetic modifications are a reminder of trained immunity.

### Rheumatoid arthritis

Rheumatoid arthritis (RA) mostly affects joints but can also cause damage to other organs like the lungs. Monocytes have been associated with RA pathophysiology (107). Monocytes from patients exhibit more proinflammatory cytokine (IL1) gene expression (108). After stimulation, they also produce more IL1β and IL6 (109). Metabolic changes toward upregulated glycolysis have been described in RA macrophages (110–112). DNA methylation changes were also found in patients after methotrexate treatment (113). Multiomics techniques will allow new development of therapeutic strategies (114). Increased proinflammatory cytokines production in monocytes, upregulated glycolysis, and methylation changes are features of trained immunity.

## Discussion

**FIGURE 2:**
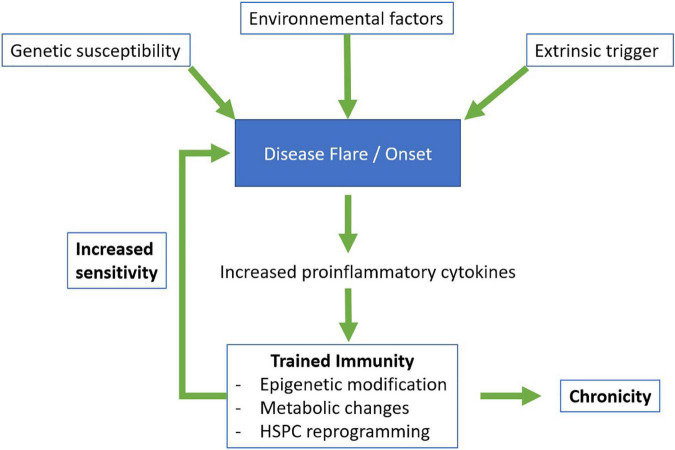
Proposed model for the involvement of trained immunity in autoinflammatory and autoimmune diseases. HSPC, hematopoietic stem and progenitor cells.

Under physiological conditions, innate and inflammatory responses are rapid and transient. However, when the system is dysregulated, these responses can be maladapted, too strong and/or persist. Trained immune cells ensure a beneficial prompt response to a second stimulation, yet we hypothesize that they could also be one of the tipping elements leading to flares and/or chronicity of immune-mediated diseases (Figure 2). Their increased responsiveness could contribute to the development and/or maintenance of chronic or recurrent inflammation and tissue destruction, which characterize autoinflammatory and autoimmune diseases. The initial stimulus leading to or fueling autoinflammatory and autoimmune diseases remain to be identified, but trained immunity can be induced by endogenous and environmental stimuli, such as oxidized low-density lipoprotein (oxLDL) and western diet (115–117), that are also known or suspected to participate to the development of autoinflammatory and autoimmune diseases (118, 119). Consequently, strategies targeting metabolism and epigenetic reprogramming, which represent the hallmarks of trained immunity, are being proposed as potential treatments or prevention strategies of autoinflammation and autoimmunity.

The expanding field of the pathophysiology of autoinflammatory diseases and autoimmune diseases is wider than ever before thanks to new technologies like single-cell RNA sequencing and high dimensional cytometry. In the study by Zhang et al. the authors studied different stimuli to induce trained immunity in monocytes/macrophages. They used unsupervised cluster analysis, to identify three distinct subsets of macrophages, equally present across the four different stimuli they used. Interestingly, one macrophage subpopulation did not express a trained immunity phenotype. The mechanism by which the differentiation did not occur is not known. Another interesting fact is that the presence or absence of lymphocytes made a difference in cytokine expression, implying an impact of cell-cell interactions, and possibly adaptive immunity cells, on trained immunity (120). The mechanisms through which trained immunity is induced are yet to be fully explained, combining single-cell level analysis with chromatin condensation analysis will be of great help. Another coupling that could be interesting is the analysis of the metabolism of the different cell subpopulations.

New pathways involved in trained immunity are being discovered regularly. IL37, a member of the IL1 family, exerts an anti-inflammatory activity (121). It has been linked to various diseases like SLE (122), arterial stiffness in Behcet’s disease (123), and inflammatory osteoarticular diseases (124). IL37 has been shown to inhibit innate immunity (125) but more recently, Cavalli et al. demonstrated that IL37 can block trained immunity changes. They used a model of *Candida albicans* infection and looked at trained immunity features like metabolism and epigenetic changes. More specifically, IL37 was responsible for the suppression of glycolysis, of HIF-1α as part of the AKT/mTOR/HIF-1α pathway, and for epigenetic changes (H3K4me3) induced by the trained immunity phenotype (126). After a growing list of stimuli of trained immunity, inhibitors are beginning to be described as well, allowing new explanations for anti-inflammatory mechanisms, and possibly new treatments (127, 128).

Among the many immune-related diseases, autoinflammatory diseases seem to be the more relevant to explore the hypothesis of a trained immunity origin, regarding their innate immunity orientation. Exciting opportunities lie ahead with possible analysis of metabolism, epigenetic modification, single-cell level analysis, and multiple cytokine assays to further characterize their pathophysiology. Trained immunity might bring answers to formerly unknown mechanisms. But at the same time, it will open up new questions regarding disease flares, pathways involved, and even diet and microbiota implications in the pathophysiology. Autoimmune diseases are not to be forgotten as we discussed in this review, even if their pathophysiology is more adaptive immunity oriented. A new chapter is starting for exploring unknown venues and unraveling more discoveries in our immune system physiology journey.

## Conclusion

There is growing evidence of the implication of the innate immune system across many autoinflammatory and autoimmune diseases. Trained immunity is not the sole responsible for their pathophysiology but an overactivation loop could participate in the occurrence of flares or chronicity. More studies are necessary to specifically explore metabolic changes, epigenetic modifications, and innate immune cell phenotypes in those diseases. This could allow new therapeutic targets and further understanding of our immune system.

## Author contributions

AS had the idea and wrote the manuscript. A-SB, CG and MM made revisions. All authors contributed to the article and approved the submitted version.
